# Immune Checkpoint Inhibitor-Induced Scleroderma-Like Pulmonary Fibrosis in a Patient With Advanced Lung Cancer: A Case Report

**DOI:** 10.7759/cureus.107642

**Published:** 2026-04-24

**Authors:** Juan O Rodriguez Padilla, Yatin Kheti, Felix Carrillo, Camila Villacreses

**Affiliations:** 1 Internal Medicine, Lakeland Regional Health, Lakeland, USA; 2 Pulmonary and Critical Care Medicine, Lakeland Regional Health, Lakeland, USA; 3 Hospital Medicine, Lakeland Regional Health, Lakeland, USA

**Keywords:** autoimmune pneumonitis, immune checkpoint inhibitors, interstitial lung disease, pembrolizumab, pulmonary fibrosis, scleroderma-like syndrome

## Abstract

We describe a 79-year-old man with stage IV non-small cell lung cancer previously treated with pembrolizumab who presented with progressive dyspnea and hypoxemia. Evaluation demonstrated bilateral ground glass opacities with interstitial changes compatible with a nonspecific interstitial pneumonia pattern, echocardiographic findings concerning for pulmonary hypertension, Raynaud phenomenon, and autoimmune serologies including elevated polymyositis/scleroderma 75 (PM/Scl 75) and small nuclear ribonucleoprotein 70 kilodalton (SNRNP 70Kd) antibodies suggestive of a scleroderma-like autoimmune process. Infectious evaluation, including blood and urine cultures and respiratory pathogen testing, did not identify a causative pathogen, although sputum culture, bronchoscopy with bronchoalveolar lavage, and lung biopsy were not performed, which is an important limitation. The patient was treated with broad-spectrum antibiotics, systemic corticosteroids, and ventilatory support, with planned initiation of additional immunosuppressive therapy. Despite these interventions, his respiratory status worsened, requiring mechanical ventilation, and his course was complicated by renal failure, heparin-induced thrombocytopenia, and multiorgan dysfunction. This case illustrates a rare, likely immune checkpoint inhibitor-associated scleroderma-like pulmonary syndrome in a patient with multiple comorbidities and underscores the need to consider progressive autoimmune disease among several potential etiologies in patients presenting with unexplained respiratory decline after immunotherapy, while acknowledging that a definitive causal relationship cannot be established in the absence of a complete diagnostic evaluation.

## Introduction

Immune checkpoint inhibitors have dramatically improved outcomes in patients with non-small cell lung cancer (NSCLC) but are associated with a broad spectrum of immune-related adverse events. Among pulmonary toxicities, checkpoint inhibitor pneumonitis (CIP) is increasingly recognized, whereas systemic sclerosis and scleroderma-like syndromes, particularly those manifesting with interstitial lung disease (ILD) and pulmonary hypertension, remain exceedingly rare and are not yet well characterized in routine practice [1]. Mechanistically, immune checkpoint inhibitors such as pembrolizumab block the programmed death 1 (PD-1) receptor, thereby removing co-inhibitory signals that normally maintain peripheral self-tolerance [2]. This disruption can unmask autoreactive T-cell populations capable of of producing an autoimmune process, including systemic sclerosis phenotypes, rather than merely causing nonspecific inflammation [3,4]. In clinical settings, differentiating immune checkpoint inhibitor-related scleroderma-like ILD from more common entities such as CIP, infection, cardiogenic pulmonary edema, chronic obstructive pulmonary disease exacerbations, and progression of underlying malignancy can be challenging because radiologic and clinical features often overlap. We report a case in which pembrolizumab therapy appears temporally associated with the emergence of a systemic scleroderma-like autoimmune process manifesting as ILD with pulmonary hypertension in a patient with multiple comorbidities and an incomplete diagnostic evaluation, emphasizing diagnostic complexity and the need for careful causal attribution.

## Case presentation

**Figure 1:**
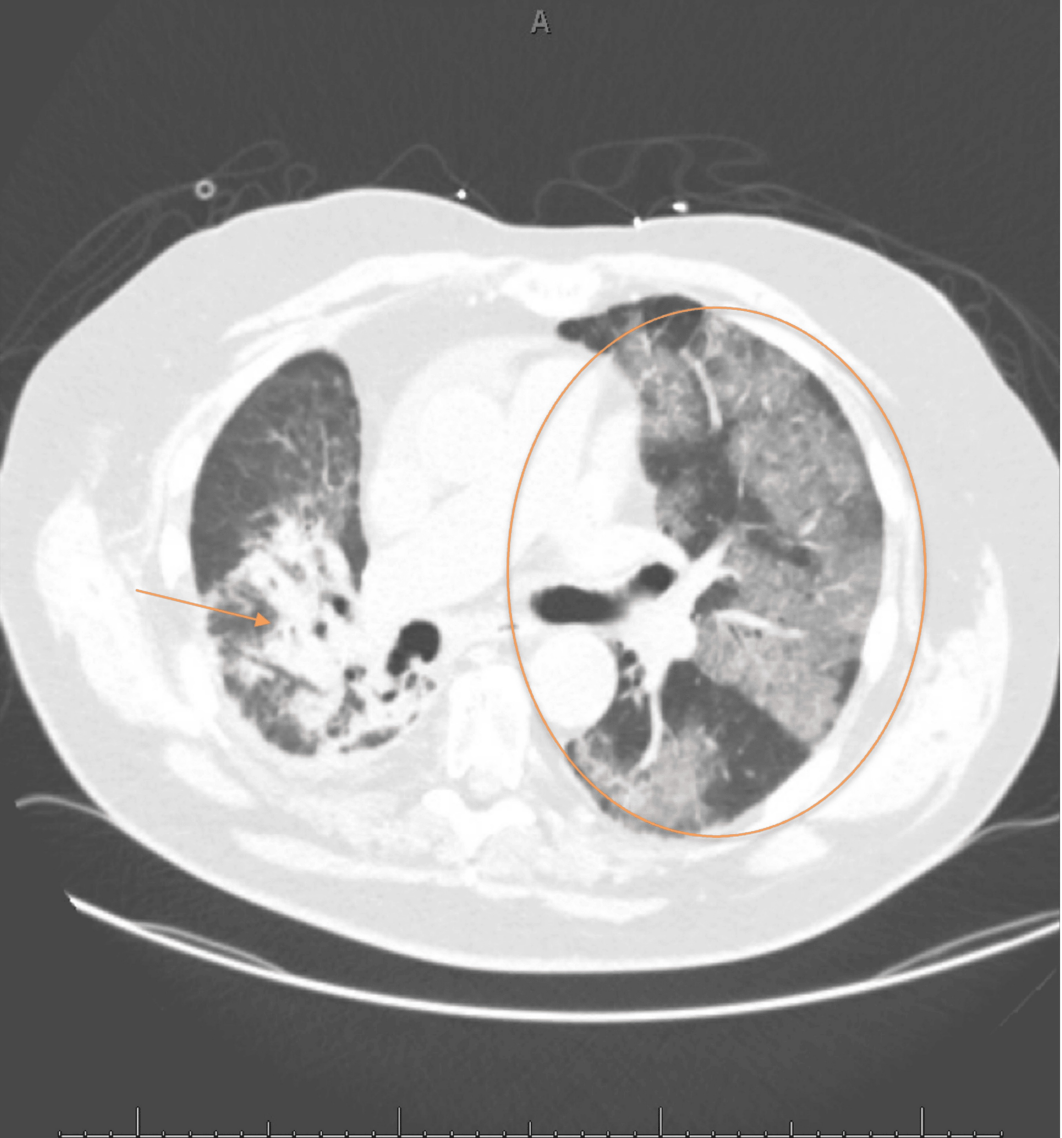
Computed tomography of the chest on admission Axial computed tomography scan of the chest demonstrating bilateral ground-glass opacities and interstitial changes (outlined area) with focal right-sided consolidation (arrow), described based on radiographic appearance as consistent with a nonspecific interstitial pneumonia pattern. Image interpretation was performed as part of routine clinical radiology evaluation without use of proprietary imaging analysis software or commercial classification tools.

The patient, a 79-year-old man with stage IV NSCLC previously treated with pembrolizumab, chronic kidney disease, and heart failure, presented with several weeks of progressive dyspnea, worsening exertional intolerance, and new resting hypoxemia. On admission, he required supplemental oxygen, and initial evaluation revealed bilateral ground-glass opacities with interstitial changes on chest computed tomography compatible with a nonspecific interstitial pneumonia pattern, in addition to chronic emphysematous changes (Figure 1). Transthoracic echocardiography demonstrated a reduced ejection fraction (40% to 45%) with regional wall motion abnormalities and elevated right-sided pressures concerning for pulmonary hypertension. Infectious evaluation, including blood and urine cultures and respiratory pathogen testing, did not identify a causative pathogen; however, sputum culture, bronchoscopy with bronchoalveolar lavage, and lung biopsy were not performed.

Initial management included broad-spectrum antibiotics, diuresis for possible heart failure exacerbation, and systemic corticosteroids for suspected inflammatory lung injury, along with escalation of oxygen therapy. Despite these interventions, the patient’s respiratory status progressively worsened, requiring escalation to high-flow nasal cannula and noninvasive ventilation via bilevel positive airway pressure. Autoimmune serologies were obtained in the context of persistent hypoxemia and a history of Raynaud phenomenon and demonstrated elevated polymyositis/scleroderma 75 (PM/Scl 75) and small nuclear ribonucleoprotein 70 kilodalton (SNRNP 70Kd) antibodies, again supporting a scleroderma-like autoimmune process (Table 1). Plans were made to initiate mycophenolate mofetil as a steroid-sparing immunosuppressive agent once corticosteroid tapering was feasible.

**Table 1:** Autoimmune serologic findings. Autoimmune serologic testing obtained during evaluation demonstrating antibody abnormalities supportive of a scleroderma-like autoimmune process. PM/Scl-75 and SNRNP 70Kd results are shown; additional serologies (including ANA and others) were obtained but complete results are not reported in this manuscript. All laboratory assays represent routine clinical testing and do not involve proprietary diagnostic scoring systems, classification tools, or licensed instruments. ANA, antinuclear antibody; PM/Scl-75, polymyositis/scleroderma 75 antibody; SNRNP 70Kd, small nuclear ribonucleoprotein 70 kilodalton.

Antibody	Result (units)	Reference range	Interpretation
PM/Scl-75	28	< 20	Mildly elevated
SNRNP 70Kd	51	< 20	Significantly elevated

By hospital day eight, renal function continued to decline (creatinine 2.67 mg/dL) (Table 2). The development of thrombocytopenia with a positive heparin-induced thrombocytopenia antibody prompted discontinuation of enoxaparin and initiation of argatroban. The patient also required transfusion of two units of packed red blood cells for symptomatic anemia (hemoglobin improved from 6.3 to 9.9 g/dL) (Table 2). Over the subsequent weeks, progressive respiratory failure with increasing oxygen requirements and worsening gas exchange led to transfer to the intensive care unit. By hospital day 30, the patient developed worsening respiratory acidosis and encephalopathy, necessitating endotracheal intubation and mechanical ventilation. Subsequent imaging revealed pneumomediastinum, and a tracheostomy was performed on hospital day 40 due to prolonged ventilator dependence.

**Table 2:** Laboratory findings with reference ranges. proBNP, pro-brain natriuretic peptide, PM/Scl-75, polymyositis/scleroderma 75 antibody; SNRNP 70Kd, small nuclear ribonucleoprotein 70 kilodalton.

Test	Patient value	Units	Reference range
White blood cell count	18.96	×10^9/L	6 - 12
Sodium	129	mEq/L	135 - 145
Creatinine (admission)	2.05	mg/dL	0.5 - 1.2
proBNP	1298	pg/mL	< 125
Troponin	30 to 34	ng/L	< 21
PM/Scl-75 antibody	28	units	< 20
SNRNP 70Kd antibody	51	units	< 20
Hemoglobin (nadir)	6.3	g/dL	11.6 - 15
Hemoglobin (post transfusion)	9.9	g/dL	11.6 - 15
Creatinine (hospital day 8)	2.67	mg/dL	0.5 - 1.2

Despite aggressive supportive care, including sedation, anticoagulation with argatroban, nutritional support, antimicrobial therapy tailored to intermittent infectious concerns, and continued corticosteroid therapy, the patient remained critically ill with persistent renal dysfunction and thrombocytopenia. His course was further complicated by hemodynamic instability requiring vasopressor support. On hospital day 45, the patient developed progressive hypotension and bradycardia, and resuscitative efforts were unsuccessful. No autopsy was performed, limiting further pathologic characterization of the pulmonary process.

This report did not use proprietary scoring systems, paid software, or licensed clinical grading tools. All diagnostic evaluations were part of routine clinical care, including computed tomography imaging, echocardiography, and standard laboratory testing. Lung cancer staging was documented using the American Joint Committee on Cancer tumor node metastasis system 4], and chronic kidney disease staging followed the Kidney Disease Improving Global Outcomes criteria [5]. Both represent publicly available clinical classification frameworks and were used only descriptively. No copyrighted calculators, proprietary radiologic analysis platforms, or commercial severity indices were applied.

## Discussion

The incidence of pulmonary fibrosis in immune checkpoint inhibitor-treated patients with anti-scleroderma antibodies remains undefined. Our literature review found no studies directly addressing this intersection. However, several retrospective and prospective studies report increased fibrosis incidence among seropositive patients. Prior studies have demonstrated that specific autoantibody profiles in systemic sclerosis are associated with higher rates of pulmonary fibrosis and worse pulmonary outcomes. Fertig et al. reported a 79% fibrosis rate in anti-U11/U12 RNP-positive systemic sclerosis patients [6]. Morrisroe et al. documented progression to pulmonary fibrosis among anti-Scl 70 and anticentromere-positive patients [7]. Guillén del Castillo et al. demonstrated worse lung function outcomes and lower survival in anti-Scl 70 patients compared to those with anti-PM/Scl antibodies [8].

Immune checkpoint inhibitors such as anti-PD-1 antibodies block co-inhibitory receptors that are essential for maintaining peripheral immune tolerance [2]. Under normal physiological conditions, the PD-1/PD-L1 axis suppresses autoreactive T-cell responses and prevents tissue damage [2]. By removing this regulatory brake, immune checkpoint inhibitors can unleash preexisting autoreactive T-cell clones and promote a loss of self-tolerance, thereby triggering de novo systemic autoimmune processes rather than simply causing nonspecific inflammation [3]. Immune checkpoint inhibitor-associated scleroderma-like presentations share some features with primary systemic sclerosis but also demonstrate key differences, including a lower frequency of Raynaud phenomenon and variable seropositivity patterns [1,3]. In the present case, the temporal relationship between pembrolizumab exposure and the emergence of scleroderma-associated autoantibodies, Raynaud phenomenon, and progressive ILD is suggestive of an immune checkpoint inhibitor-triggered autoimmune etiology, although causality cannot be definitively established [1-3,6-8].

The incidental identification of chronic hepatitis C virus infection during this hospitalization introduces an important diagnostic consideration. Hepatitis C virus is associated with extrahepatic autoimmune manifestations, including mixed cryoglobulinemia, which can produce pulmonary and renal complications through vascular and interstitial deposition of cryoglobulins [9]. However, the serologic profile in this patient, with elevated PM/Scl 75 and SNRNP 70Kd antibodies, is characteristic of scleroderma overlap syndromes rather than hepatitis C virus-associated cryoglobulinemia. Cryoglobulin testing and hepatitis C viral load were not obtained, which represents a limitation of this report.

The development of heparin-induced thrombocytopenia during hospitalization represents an additional complicating factor. Heparin-induced thrombocytopenia is a prothrombotic disorder that can contribute to multiorgan dysfunction through microvascular and macrovascular thrombosis, independent of the underlying autoimmune process [3]. Although the patient’s progressive respiratory failure and ILD were likely influenced by an autoimmune process, the concurrent heparin-induced thrombocytopenia, heart failure, chronic kidney disease, and the need for argatroban anticoagulation may have contributed to the hemodynamic instability and multiorgan failure observed in the terminal phase [3,5]. Distinguishing autoimmune-mediated organ injury from infection, heart failure, and heparin-induced thrombocytopenia-related thrombotic complications is clinically challenging, and several mechanisms may have been active in parallel [3].

Overall, the patient’s rapid pulmonary decline, high oxygen requirements, changes on imaging compatible with a nonspecific interstitial pneumonia pattern, and serologic evidence of a scleroderma spectrum process in the context of prior pembrolizumab exposure point toward a diagnosis of immune checkpoint inhibitor-associated autoimmune ILD mimicking systemic sclerosis [1-3,6-8]. However, in the absence of histopathologic confirmation, complete infectious and rheumatologic testing, and longitudinal pre-treatment serologies, this case can only support a strong suspicion of an association between pembrolizumab therapy and the observed scleroderma-like pulmonary fibrosis, rather than establishing a direct causal relationship. Despite high-dose corticosteroids and planned immunosuppression, his condition progressed to multiorgan failure.

Limitations

This case report has several important limitations that constrain causal inference and generalizability. First, bronchoscopy with bronchoalveolar lavage, surgical lung biopsy, and cryoglobulin studies were not performed, limiting the ability to exclude alternative etiologies such as infection, drug-induced lung injury unrelated to immune checkpoint blockade, or hepatitis C-associated pulmonary involvement. Second, the patient’s multiple comorbidities, including chronic heart failure, chronic kidney disease, chronic obstructive pulmonary disease, and heparin-induced thrombocytopenia, likely contributed to respiratory decline and multiorgan failure and made it difficult to isolate the impact of a putative autoimmune process. Third, the lack of baseline autoimmune serologies before pembrolizumab initiation prevents determination of whether the detected autoantibodies were truly de novo or represented an unrecognized preexisting condition that later manifested clinically. Finally, the absence of autopsy findings precludes detailed pathologic characterization of pulmonary and vascular changes. These limitations highlight the need for cautious interpretation of the observed temporal association and underscore the value of systematic diagnostic workup in future similar cases.

This case underscores the growing recognition of systemic autoimmune complications of immune checkpoint inhibitors and the need for early consideration of rheumatologic etiologies when encountering atypical or refractory respiratory symptoms after immunotherapy, while maintaining caution regarding causal attribution in the setting of an incomplete diagnostic evaluation and multiple confounders. Further studies are needed to quantify the risk of fibrosis among seropositive patients receiving immunotherapy and to define optimal management strategies.

## Conclusions

Immune checkpoint inhibitors can trigger rare but life-threatening autoimmune syndromes, including scleroderma-like ILD with pulmonary hypertension. Clinicians should maintain a high index of suspicion in patients with prior immune checkpoint inhibitor therapy and evolving respiratory symptoms, while systematically evaluating for alternative causes such as infection, heart failure, chronic obstructive pulmonary disease exacerbation, and recurrent pneumonitis. Early serologic workup and multidisciplinary management involving oncology, pulmonology, rheumatology, and critical care may help identify emerging autoimmune complications and guide individualized treatment. In cases with incomplete diagnostic evaluation and multiple comorbidities, conclusions should emphasize a probable association rather than definitive causation and highlight diagnostic uncertainty. Further studies are needed to quantify the risk of fibrosis among seropositive patients receiving immunotherapy and to define optimal management strategies.
